# Potentiation of cognitive enhancer effects of Alzheimer’s disease medication memantine by alpha7 nicotinic acetylcholine receptor agonist PHA-543613 in the Morris water maze task

**DOI:** 10.1007/s00213-021-05942-4

**Published:** 2021-08-13

**Authors:** Nóra Bruszt, Zsolt Kristóf Bali, Sai Ambika Tadepalli, Lili Veronika Nagy, István Hernádi

**Affiliations:** 1 János Szentágothai Research Center, Center for Neuroscience, University of Pécs, 20 Ifjúság str, 7624 Pécs, Hungary; 2 Institute of Physiology, Medical School, University of Pécs, 12 Szigeti str, 7624 Pécs, Hungary; 3 Grastyán Endre Translational Research Center, University of Pécs, 6 Ifjúság str, 7624 Pécs, Hungary; 4 Department of Experimental Zoology and Neurobiology, Faculty of Sciences, University of Pécs, 6 Ifjúság str, 7624 Pécs, Hungary

**Keywords:** Spatial learning, Long-term memory, Dementia, Scopolamine, Combined treatment, Behavior

## Abstract

**Rationale:**

There are controversial pieces of evidence whether combination therapies using memantine and cholinesterase inhibitors are beneficial over their monotreatments. However, results of preclinical studies are promising when memantine is combined with agonists and allosteric modulators of the alpha7 nicotinic acetylcholine receptor (nAChR).

**Objectives:**

Here, we tested the hypothesis that cognitive enhancer effects of memantine can be potentiated through modulating alpha7 nAChRs in a scopolamine-induced amnesia model.

**Methods:**

Monotreatments, as well as co-administrations of selective alpha7 nicotinic acetylcholine receptor agonist PHA-543613 and memantine were tested in the Morris water maze task in rats. The efficacy of the co-administration treatment was observed on different domains of spatial episodic memory.

**Results:**

Low dose of memantine (0.1 mg/kg) and PHA-543613 (0.3 mg/kg) successfully reversed scopolamine-induced short-term memory deficits both in monotreatments and in co-administration. When recall of information from long-term memory was tested, pharmacological effects caused by co-administration of subeffective doses of memantine and PHA-543613 exceeded that of their monotreatments.

**Conclusion:**

Our results further support the evidence of beneficial interactions between memantine and alpha7 nAChR ligands and suggest a prominent role of alpha7 nAChRs in the procognitive effects of memantine.

**Supplementary Information:**

The online version contains supplementary material available at 10.1007/s00213-021-05942-4.

## Introduction

Alzheimer’s disease (AD) is a progressive neurodegenerative disorder characterized by irreversible cognitive decline, which affects millions of people over the age of 65 years worldwide (Gaugler et al. 2016). The currently approved medications for the treatment of AD (acetylcholinesterase inhibitors such as donepezil and galantamine, and the non-competitive NMDA receptor antagonist memantine) provide statistically significant but clinically limited improvements in symptoms associated with behavior, cognition, and global mental function (Dou et al., 2018; Raina et al., 2008; Tsoi et al., 2016). Therefore, the development of novel treatment strategies is crucial in the field. There is an increasing interest in combination therapies which target different signaling pathways involved in learning and memory and in the progression of AD (Parsons et al. 2013). Although a fixed-dose combination of donepezil and memantine has already been licensed and marketed for AD, there is still a lack of clear clinical evidence of additional benefits of the donepezil-memantine combination treatment over the corresponding monotherapies (Deardorff & Grossberg, 2016; Farrimond et al., 2012; Molino et al., 2013; Tsoi et al., 2016; Tsoi et al., 2016). In line with this, some preclinical studies have also found poor efficacy of donepezil-memantine co-administration in different rodent models. For example, donepezil-memantine combination treatment failed to enhance memory performance of rats in the radial arm maze task (Wise and Lichtman 2007), as well as in an associative learning task in aged rabbits (Woodruff-Pak et al. 2007). However, a greater efficacy has been observed in the same study when memantine was simultaneously administered with galantamine instead of donepezil (Woodruff-Pak et al. 2007). Superiority of galantamine-memantine combination over donepezil-memantine combination seems to be related to the additional action of galantamine on alpha7 nicotinic acetylcholine receptors (nAChR) besides its cholinesterase inhibitor effect (Koola et al. 2018). Consequently, alpha7 nAChRs may be of predominant importance in the potentiation of the effects of memantine.

In a previous study from our laboratory, we tested whether pharmacological interaction between a selective alpha7 nAChR agonist PHA-543613 and memantine may have beneficial effects on working memory performance of rats in the spontaneous alternation task over their monotreatments (Bali et al. 2019a). Our previous results indicate a clear additive interaction in the behavioral effects of the two compounds suggesting a potential novel preclinical experimental approach for the treatment of AD. In the present study we further tested the interactions between PHA-543613 and memantine on cognitive functions, including spatial learning, short-term and long-term memory in the Morris water maze task using the scopolamine-induced transient amnesia model in rats.

## Materials and Methods

### Animals

Adult male Long Evans rats (Charles River Laboratories, Calco, Italy), 6–8 months old, and weighing 350–500 g were applied in the current study. Dose–response curves for memantine and PHA-543613 were assessed in altogether 72 rats, whereas 65 other rats were used in the investigation of the effectiveness of co-administration treatments. Animals were pair-housed under 12/12 h light/dark cycle with controlled temperature and humidity in the animal house of the Szentágothai Research Centre, University of Pécs, Hungary. In the animal house, the lights were ON from 7 a.m. to 7 p.m., and the animals were tested in the light period. Rats were fed daily with 17 g/animal of laboratory chow to prevent obesity and related health problems (e.g., cardiovascular and renal diseases). Water was available ad libitum. The experiments were approved by the Animal Welfare Committee of the University of Pécs, and the National Scientific Ethical Committee on Animal Experimentation (ÁTET) at the Ministry of Agriculture (license no.: BA02/2000–25/2015). All procedures fully complied with the Decree No. 40/2013. (II. 14.) of the Hungarian Government and the EU Directive 2010/63/EU on the protection of animals used for scientific purposes.

### Morris water maze paradigm for assessing spatial long-term memory

The protocol of the Morris water maze (MWM) navigation task was the same as described in a previous paper from our laboratory by Tadepalli et al. (2019). Experiments were carried out in a blue circular pool with 180 cm in diameter and 90 cm in height, filled to the depth of 30 cm of blue painted, blurred water (mixing 500 g milk-powder and 30 ml blue food coloring). The area of the pool was divided into four virtual quadrants (NW, SW, SE, NE). The experimental protocol consisted of four training days and an additional probe trial day. During the training days a platform was placed in the center of the SW quadrant (submerged 1 cm below the surface), and the rats had to learn the location of the hidden platform with the help of visual cues placed around the maze. All animals had to perform four trials per training day. In each trial, the rats were placed into different quadrants changed clockwise, and were allowed to search for the hidden platform for 2 min. During the trials, the escape latency (the time until the platform was found), the swimming path length to the platform and the swimming speed of the animals were measured and analyzed using Ethovision XT10 software (Noldus, Wageningen, Netherlands). If the platform was not found during the trial, rats were put on the platform for 10 s, and 2 min was recorded as escape latency. The performance of the animals on the first training day was used to evaluate their short term memory performance. On the fifth day, a single probe trial was performed for testing the recall of long-term memory. The platform was removed from the pool and the animals were allowed to search the pool for 2 min. During the probe trial, the time spent in the target quadrant (the quadrant of the missing platform) was measured as an index of long-term memory recall.

### Drugs and routes of administration

Scopolamine hydrobromide (Cat. No. 1414, Tocris), PHA-543613 hydrochloride (Cat. No. 3092, Tocris), and memantine hydrochloride (Cat. No. 0773, Tocris) were dissolved in physiological saline (vehicle) to create a final injection volume of 1 ml/kg. On each training day, scopolamine was injected intraperitoneally (IP) 15 min before the experimental sessions, while PHA-543613 and/or memantine were injected subcutaneously (SC) 45 min before the sessions (i.e., 30 min before scopolamine administration). When mono-treatments were tested, drugs that were not administered were replaced with the vehicle. On the fifth day (probe trial day), pharmacological treatments were not applied.

### Experimental design

The first series of experiments were established for the determination of the dose–response relationships for memantine and PHA-543613. Memantine was administered in the doses of 0.1 mg/kg, 0.3 mg/kg and 1.0 mg/kg (Mem0.1, Mem0.3, and Mem1.0, respectively). PHA-543613 was administered in the doses of 0.3 mg/kg, 1.0 mg/kg and 3.0 mg/kg (PHA0.3, PHA1.0, and PHA3.0, respectively). Efficacy of the applied drugs in different doses was tested on cognitive performance of the rats using the scopolamine-induced (0.1 mg/kg, Scop) transient amnesia model. The monotreatments with different doses of memantine and PHA-543613 were compared to the same vehicle-treated and scopolamine-treated groups. In the second series of experiments, pharmacological interactions between subeffective doses of memantine (0.1 mg/kg) and PHA-543613 (0.3 mg/kg) and the cognitive enhancer effects of their co-administration were tested and were compared with the effects of monotreatments. Animals received the following treatments: vehicle alone (VEH), scopolamine alone (Scop), memantine monotreatment in 0.1 mg/kg dose followed by scopolamine (Mem0.1), PHA-543613 monotreatment in 0.3 mg/kg dose followed by scopolamine (PHA0.3), and co-administration treatment followed by scopolamine (Mem0.1&PHA0.3). Treatments were applied in a between-subject design (different treatments were given to different groups of subjects).

### Statistical analysis

Data were expressed as mean ± standard error of the mean (SEM). Statistical analyses were performed using the IBM SPSS 20.0 software. The performance of the animals during the training phase was analyzed by mixed-ANOVA with DAYs and TREATMENTs as factors. Since improvement in task performance was most obvious in the first two days of the training, the changes of escape latency and swimming path length were further analyzed trial-wise within the first and the second training days. When significant interactions were found, interaction contrasts were tested between specific pairs of treatments and specific pairs of training sessions (i.e. days or trials). The interaction contrasts tested the effects of a given treatment (in reference to the scopolamine-only treatment) on the change of performance sequentially from one session to the next and from the 1st to the last (4th) session (e.g. [Control vs. Scop] × [day1 vs. day2], [PHA0.3 vs. Scop] × [trial3 vs trial4], etc.).

For analyzing probe trial data, univariate ANOVA test was applied followed by post-hoc LSD-test. In all statistical analyses, *p* < 0.05 was considered significant.

## Results

### Monotreatments with memantine and PHA-543613 in different doses

**Fig. 1:**
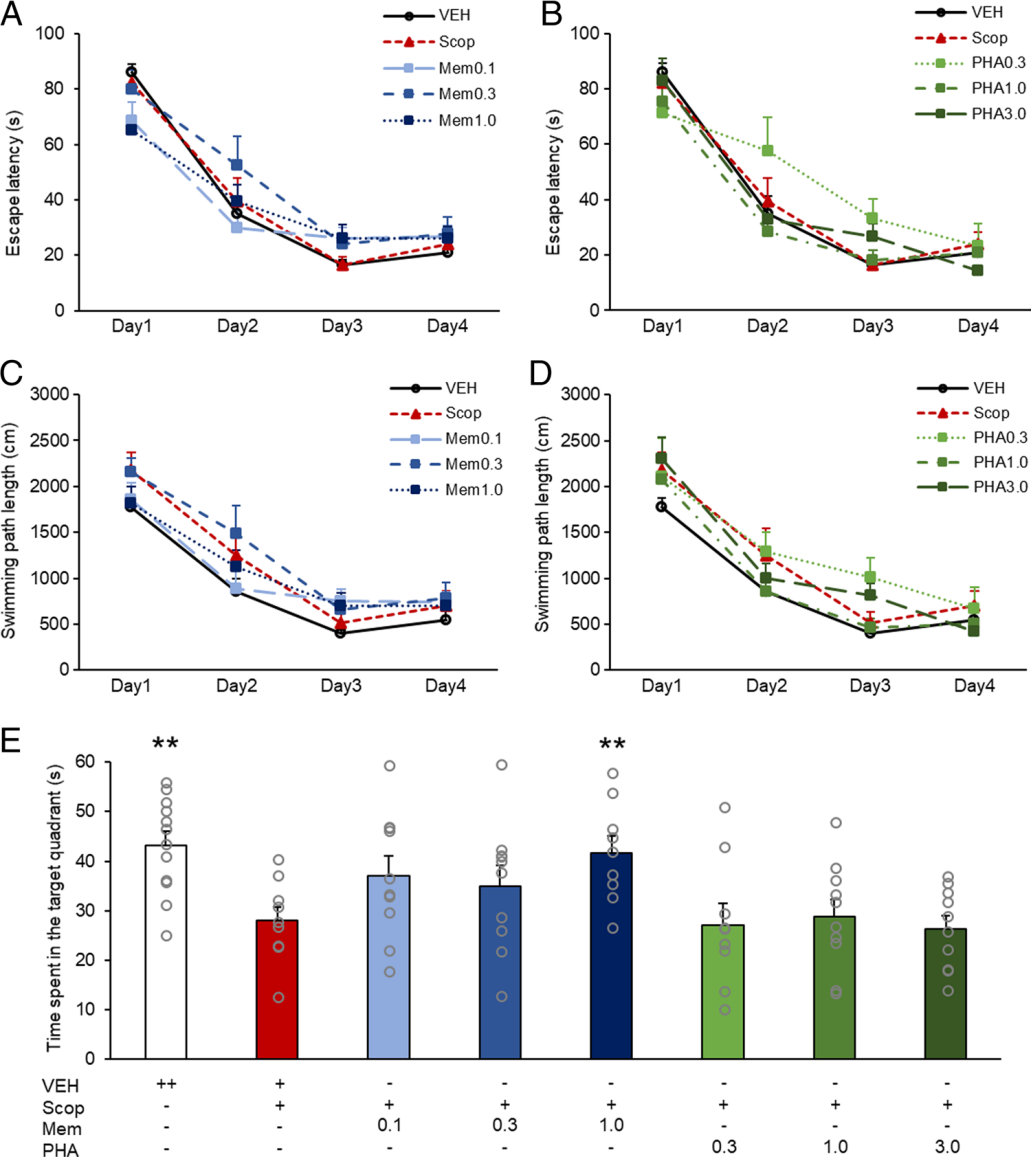
Dose–response relationship for the effects of memantine and PHA-543613 on spatial learning and recall of long-term memory in the water maze task. Overall escape latency (**A**-**B**) and swimming path length (**C**-**D**) during the training days (days 1–4) showed no effect of the treatments. Time spent in the target quadrant in the probe trial (day 5) showed significant main effect of the treatments (**E**). Data are expressed as mean + SEM. Grey circles on the bar chart represent individual data points. Significant differences between a given treatment and the scopolamine-only treatment were marked with asterisks: **p < 0.01

First, the dose–effect relationships of memantine and PHA-543613 monotreatments were analyzed. During the training phase (days 1–4), the mean escape latency of the animals significantly decreased (DAY: F(3, 213) = 177.313, *p* < 0.001) (Fig. 1A-B). There was no significant difference in the overall escape latency between vehicle, scopolamine, memantine (0.1–1.0 mg/kg) and PHA-543613-treated groups (TREATMENT: F(7, 71) = 0.845, *p* = 0.554). However, a significant interaction was found between TREATMENT and DAY (F(21, 213) = 1.918, *p* = 0.011), indicating that escape latency differentially changed in the different treatment groups over the course of the training. Contrast analysis of the interaction revealed significant differences between Scop and PHA0.3 treatment groups in the change of escape latency from the 1st to the 2nd training day ([PHA0.3 vs. Scop] × [day1 vs. day2]: − 29.0 ± 13.4 (contrast estimate ± SEM); *p* = 0.034). Nevertheless, no significant difference was found between the effect of Scop and PHA0.3 on the change of escape latency between the 1st and the last (4th) training days ([PHA0.3 vs. Scop] × [day1 vs. day4]: − 10.0 ± 12.3; *p* = 0.421) indicating that decrease of escape latency was overall similar in the two groups over the course of the whole training procedure.

Learning performance of the animals was analyzed also in terms of swimming path length which showed a profound decrease over the training days (DAY: F(3, 213) = 153.072, *p* < 0.001) (Fig. 1C-D), and significant effect of treatments on the learning progress was not revealed (TREATMENT: F(7, 71) = 1.281, *p* = 0.272) similarly to results based on escape latency. Furthermore, no significant interaction was found between TREATMENT and DAY (F(21, 213) = 1.172, *p* = 0.278) indicating that the above reported interaction in escape latency could probably be accounted to swimming speed differences. In the probe trial (Fig. 1E), the time spent in the target quadrant represented long-term memory. In contrast with the results on the training days, in the probe trial, a significant main effect of pharmacological treatments was detected (TREATMENT: F(7, 71) = 3.917, *p* = 0.001). Results showed that scopolamine-treated animals spent significantly less time in the target quadrant than the vehicle-treated group (Control vs. Scop: 43.2 ± 2.8 s vs. 28.0 ± 2.8 s, *p* = 0.002). Although memantine in lower doses (0.1 mg/kg and 0.3 mg/kg) did not improve long-term memory against scopolamine, the highest memantine dose (1.0 mg/kg) successfully reversed the memory deteriorating effect of scopolamine (Scop vs Mem1.0: 28.0 ± 2.8 s vs 41.7 ± 3.3 s, *p* = 0.009). However, PHA-543613 did not alleviate scopolamine-induced memory deficits in the applied doses.

Swimming speed was also considered as a non-cognitive measure (Singh et al. 2016) to control the possible side-effects of pharmacological treatments (Fig. S1A and B). A significant main effect of treatments was found on the swimming speed (TREATMENT: F(7, 71) = 3.289, *p* = 0.004). Post-hoc analysis showed that scopolamine increased the swimming speed in all treatment groups compared to animals in the control group reflecting that scopolamine alone increased the swimming speed of the animals (Control vs. Scop: 24.1 ± 0.5 cm/s vs. 27.7 ± 0.7 cm/s, *p* = 0.001), which was not further affected by treatments with memantine or PHA-543613 (Mem0.1: 26.7 ± 0.5 cm/s; Mem0.3: 26.5 ± 0.7 cm/s; Mem1.0: 25.8 ± 0.4 cm/s; PHA0.3: 27.9 ± 0.5 cm/s; PHA1.0: 25.8 ± 0.5 cm/s; PHA3.0: 28.3 ± 0.5 cm/s). Significant main effect of training (DAY: F(3, 213) = 8.796, *p* < 0.001) was also found without an interaction between TREATMENT and DAY (F(21, 213) = 1.147, *p* = 0.302), which suggests that the swimming speed of the animals increased independently from treatment during repeated testing.

### Co-administration of memantine and PHA-543613

**Fig. 2:**
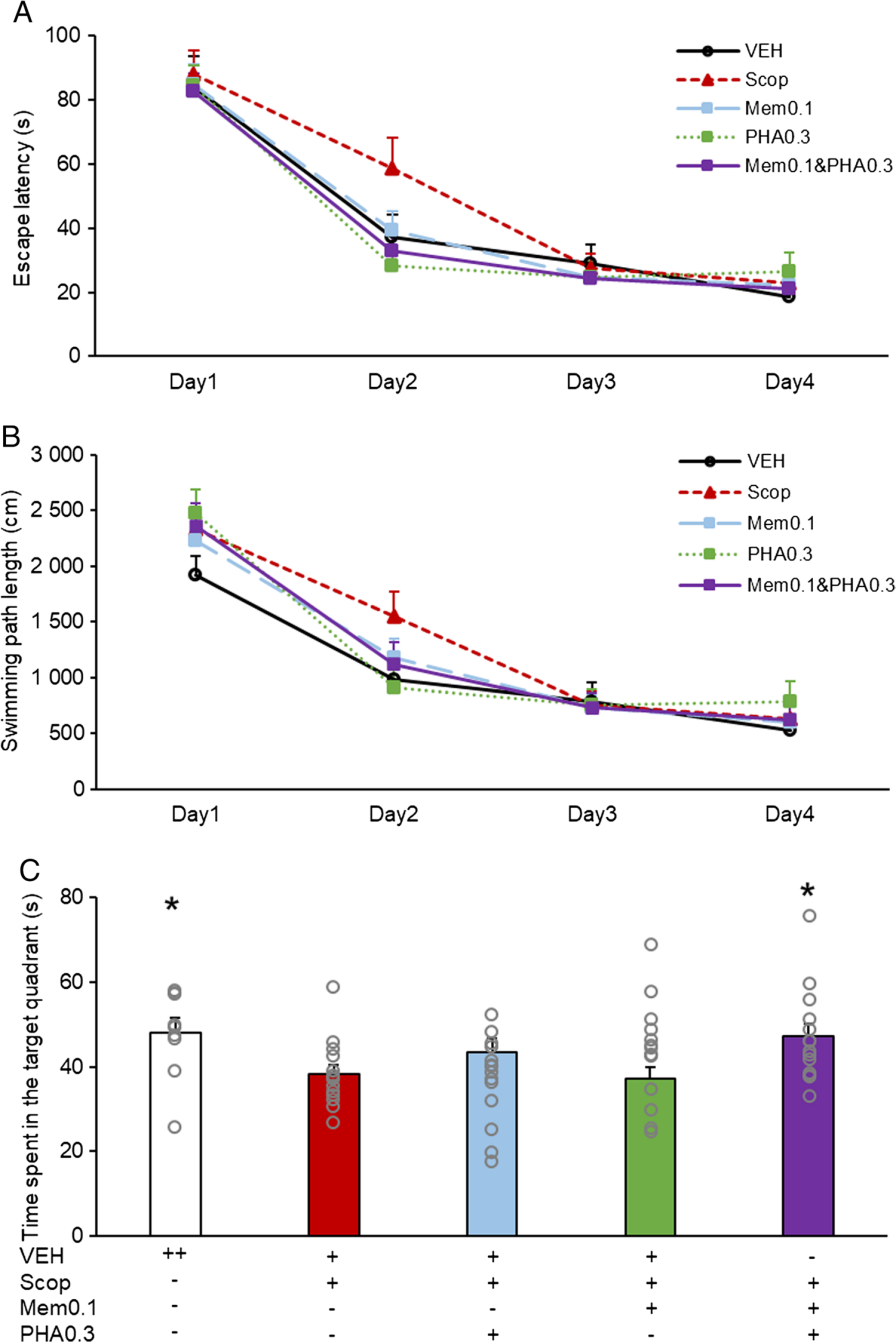
Effects of co-administration of subeffective dose of memantine and PHA-543613 on spatial learning and recall of long-term memory. Overall escape latency (**A**) and swimming path length (**B**) during the training days (days 1–4) showed no effect of the treatments. Time spent in the target quadrant in the probe trial (day 5) (**C**) showed significant main effect of the treatments. Data are expressed as mean + SEM. Grey circles on the bar chart represent individual data points. Significant differences between a given treatment and the scopolamine-only treatment were marked with asterisks: *p < 0.05

In the next experiment, a subeffective dose of memantine (0.1 mg/kg) was co-administered with a subeffective dose of PHA-543613 (0.3 mg/kg). Doses were chosen on the basis of the probe trial in the dose–response experiments. The memory enhancer effect of the co-treatment was compared with the monotreatments using the same doses of PHA-543613 and memantine. In the learning phase (days 1–4), a significant main effect of training was found, as both the escape latency (Fig. 2A) and the swimming path length (Fig. 2B) significantly decreased during the four days (DAY: escape latency: F(3, 180) = 133.533, *p* < 0.001; swimming path length: F(3, 180) = 110.325, *p* < 0.001). Neither monotreatments, nor co-administration of PHA-543613 and memantine exerted a significant main effect on escape latency (TREATMENT: F(4, 60) = 1.144, *p* = 0.345) or swimming path length (TREATMENT: F(4, 60) = 0.426, *p* = 0.789) similarly to the previous experiments with different memantine and PHA-543613 doses. Furthermore, the treatments did not significantly affect the effect of training on performance (TREATMENT × DAY: escape latency: F(12, 180) = 1.149; *p* = 0.323; swimming path length: F(12, 180) = 1.246; *p* = 0.255).

**Fig. 3:**
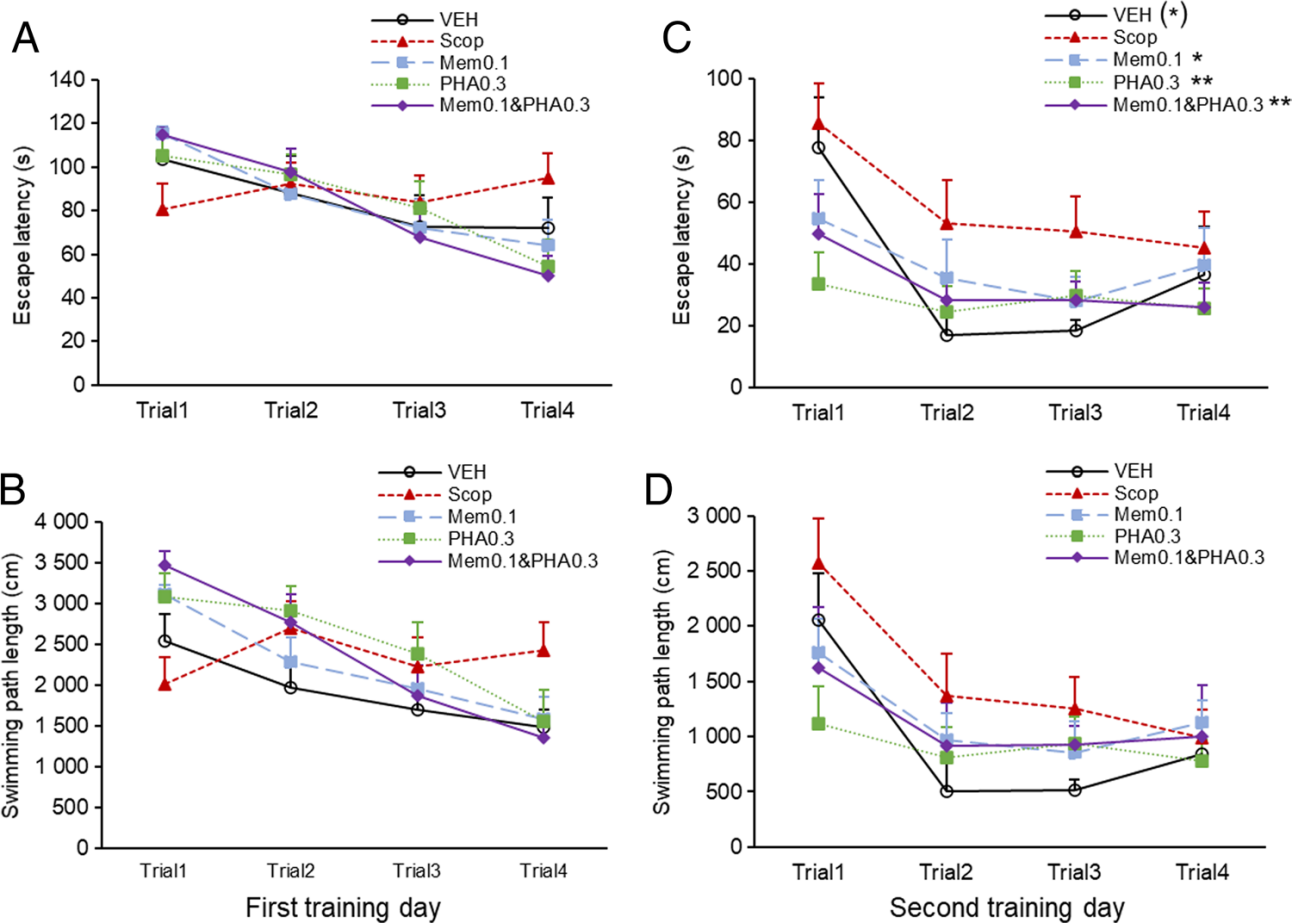
Effects of co-administration of subeffective dose of memantine and PHA-543613 on short-term memory. Escape latency (**A**) and swimming path length (**B**) on the first training day showed interaction between consecutive trials and treatments. Improvement between the first and fourth trials was significantly larger in all treatment groups compared to the scopolamine-only group. On the second training day, treatments exerted significant effects on escape latency (**C**). Swimming path length (**D**) on the second training day showed no effects of treatments. Data are expressed as mean + SEM. Significant differences between a given treatment and the scopolamine-only treatment were marked with asterisks: ^(^*^)^*p* = 0.054, *p < 0.05, **p < 0.01

Short-term memory of the animals was also analyzed which was based on the performance of the animals on the first training day (Fig. 3A, B). Results showed a significant interaction between treatments and trials on the first training day, thus treatments significantly affected the performance during training (TREATMENT × TRIAL: escape latency: F(12, 180) = 1.952, *p* = 0.031; swimming path length: F(12, 177) = 2.305, *p* = 0.009). While almost all experimental groups showed gradual improvement during the four training trials on the first day, animals treated with scopolamine-only did not learn the location of the platform.

Contrasts between pairs of treatment groups and the first and last trials (trial1 vs. trial4) on the first day were also analyzed, and results showed that changes in escape latency and swimming path length were significantly different in the group treated with scopolamine only compared to the control group ([Control vs. Scop] × [trial1 vs. trial4]: escape latency: 45.7 ± 18.4 (contrast estimate ± SEM), *p* = 0.016; swimming path length: 1475.0 ± 515.7, *p* = 0.006). These results indicate that scopolamine predominantly impaired the short-term component of spatial episodic learning. Furthermore, the scopolamine-induced learning deficit was significantly improved by memantine and PHA-543613 both in monotreatments and in co-administration of the same doses ([Scop vs. Mem0.1] × [trial1 vs. trial4]: escape latency: 65.4 ± 16.3, p < 0.001; swimming path length: 1939.4 ± 456.2, p < 0.001; [Scop vs. PHA0.3] × [trial1 vs. trial4]: escape latency: 65.3 ± 16.3, *p* < 0.001; swimming path length: 1884.0 ± 464.9, *p* < 0.001; [Scop vs. Mem0.1&PHA0.3] × [trial1 vs. trial4]: escape latency: 79.0 ± 16.3; *p* < 0.001; swimming path length: 2524.0 ± 456.2, *p* < 0.001).

Consequently, rats treated only with scopolamine still showed poor performance on the second training day (Fig. 3C, D). Here, a significant main effect of pharmacological treatments was found on escape latency on the second training day (TREATMENT: F(4, 60) = 2.932, *p* = 0.028) but not on path length (TREATMENT: F(4, 60) = 1.661, *p* = 0.171). Pairwise comparisons of escape latency data showed that control animals and animals who received the low memantine dose and low PHA-543613 dose in monotreatments or in co-administration performed better than rats treated with scopolamine only (control vs. Scop: 37.4 ± 6.7 s vs. 58.6 ± 9.4 s, *p* = 0.054; Scop vs. Mem0.1: 58.6 ± 9.4 s vs 39.3 ± 6.0 s, *p* = 0.048; Scop vs. PHA0.3: 58.6 ± 9.4 s vs. 28.3 ± 6.2 s, *p* = 0.002; Scop vs. Mem0.1&PHA0.3: 58.6 ± 9.4 s vs. 33.0 ± 5.5 s, *p* = 0.009). Here, no significant interaction was found between groups and trials (TREATMENT × TRIAL: escape latency: F(12, 180) = 1.020, *p* = 0.432; swimming path length: F(12, 180) = 0.852, *p* = 0.597), indicating that scopolamine-treated animals started to show a tendency of learning the platform location on the second training day.

In the probe trial (Fig. 2C), pharmacological treatments revealed a significant main effect on the time spent in the target quadrant (TREATMENT: F(4, 60) = 2.805, *p* = 0.033). According to post-hoc analysis, the scopolamine-treated group spent less time in the target quadrant compared to the vehicle-treated control group (Control vs. Scop: 47.9 ± 3.5 s vs. 38.3 ± 2.1 s, *p* = 0.035) and subeffective doses of memantine and PHA-543613 in monotreatments did not attenuate scopolamine-induced amnesia (Scop vs Mem0.1: 38.3 ± 2.1 s vs 37.1 ± 2.8 s, *p* = 0.766; Scop vs PHA0.3: 38.3 ± 2.1 s vs. 43.3 ± 3.2 s, *p* = 0.210). However, the co-administration treatment with memantine and PHA-543613 effectively reversed the scopolamine-induced long-term memory deficit (Scop vs. Mem0.1&PHA0.3: 38.3 ± 2.1 s vs. 47.1 ± 2.9 s, *p* = 0.029), suggesting a beneficial interaction between memantine and the alpha7 nicotinic acetylcholine receptor agonist PHA-543613.

Swimming speed was also affected in the co-administration study by both the training (DAY: F(3, 180) = 10.093, *p* < 0.001) and the treatments (TREATMENT: F(4, 60) = 3.496, *p* = 0.012). Furthermore, the treatments significantly affected the change in swimming speed over the course of the training (TREATMENT × DAY: F(12, 180) = 1.951, *p* = 0.031). Contrast analysis revealed that the change of swimming speed from the 2nd to the 3rd training days was marginally significantly larger in the Control group than in the scopolamine-only treated group [Control vs. Scop] × [day2 vs. day3]: − 3.2 ± 1.7; *p* = 0.065). As a result, the swimming speed of rats in the Control group on the last training day was similar to the groups treated with scopolamine (Fig. S1C).

## Discussion

Here, we tested the hypothesis whether the co-administration of a selective alpha7 nAChR agonist PHA-543613 increases the cognitive enhancer effects of the NMDA-receptor antagonist memantine on spatial learning and short- and long-term memory performance in the Morris water maze task.

First, average escape latency and swimming path length data over the whole training period (days 1–4) showed that all groups – irrespective of the applied treatments – improved in finding the platform, suggesting the acquisition of sufficient spatial learning by the end of the training period even in the scopolamine-only treated group. It is reasonable to conclude that spatial navigation in a stressful situation (i.e., swimming for escaping the water) is such an immanent ability of rats that is fairly resistant to the relatively weak blockade of cholinergic neurotransmission. On the first training day, scopolamine increased the locomotor activity of the animals which was assessed by the increase of their swimming speed. Therefore, we also analyzed the length of the swimming path which measure yielded similar results as the analysis of escape latency.

Nevertheless, pharmacological treatments affected spatial short-term memory performance of task-naïve animals assessed on the very first MWM training day. Animals treated with scopolamine alone consequently failed to improve in finding the platform in the subsequent trials of the first training day. This learning impairment was effectively reversed by both the monotreatments and the co-administration of subeffective doses of memantine and PHA-543613 showing significant cognitive enhancement in both conditions. In addition, the 5th day probe trial performance – which is considered as a measure of the recall of long-term memory information (Vorhees and Williams 2006) – was also significantly impaired by scopolamine, and was only enhanced by the co-administration of memantine and PHA-543613, while the monotreatments with the same low doses were completely ineffective. Thus, the effects of the co-administration treatment were superior over the monotreatments in the enhancement of the recall of spatial long-term memory indicating a beneficial interaction between memantine and alpha7 nAChR agonist PHA-543613.

Although there are contradictory pieces of evidence about the beneficial effects of combination therapies using memantine and acetylcholinesterase enzyme inhibitors over their corresponding monotherapies (Alzheimer’s Association 2016), recent preclinical findings using the combinations of memantine and alpha7 nAChR ligands seem to converge to more promising results in terms of cognitive enhancer effects. For example, in a previous research, combination treatment with low doses of galantamine and memantine significantly enhanced the performance of mice in the spontaneous alternation and object recognition tasks (Busquet et al. 2012). In a different study by Nikiforuk et al. (2016), the observed interaction of galantamine with memantine was more likely dependent on the alpha7 nAChR PAM activity of galantamine than on its acetylcholinesterase blocking activity, as the observed memory-enhancing effects of galantamine-memantine co-administration were blocked by the alpha7 nAChRs antagonist methyllycaconitine. Furthermore, in the same study, it was also demonstrated that cognitive flexibility and recognition memory were enhanced in rats when memantine was combined with galantamine or selective alpha7 nAChR PAMs (CCMI or PNU-120596) (Nikiforuk et al. 2016). In agreement with these results, in a more recent study from the same laboratory, the authors confirmed that alpha7 nAChR PAMs increase pro-cognitive effects of memantine in an object recognition memory task (Potasiewicz et al. 2020). In addition, in our previous study we also reported the improvement of spatial working memory of rats by the co-administration of memantine and the alpha7 nAChR agonist PHA-543613 in the spontaneous alternation (spatial working memory) task (Bali et al. 2019a). The above findings convergently suggest that the observed combination effects may well involve alpha7 nAChRs as common targets of memantine and PHA-543613.

The mechanisms underlying the interaction between alpha7 nAChR compounds and memantine are not yet clarified. It is well documented that memantine blocks tonic (potentially harmful) influx of Ca^2+^ ions through the NMDA receptor channel by the non-competitive antagonism of the NMDA receptor (Parsons et al. 2007). However, in an in vitro study it was demonstrated that memantine has an additional antagonist effect on alpha7 nAChRs, and that the affinity of memantine is actually higher for alpha7 nAChRs than for NMDA receptors (Aracava et al. 2005). Although the alpha7 nAChR antagonist effect of memantine seems to be difficult to relate to cognitive enhancement, recent pieces of evidence suggest that the inhibition of alpha7 nAChR-mediated activity may provide beneficial effects on neuronal function and cognition (Aracava et al. 2005). In addition, Ferchmin et al. 2003 demonstrated that the alpha7 nAChR antagonist methyllycaconitine (MLA) – similarly to nicotine – ameliorates NMDA-induced neurotoxicity in hippocampal slices. Elsewhere, it was shown that MLA was also protective against beta-amyloid-induced neurotoxicity (Martin et al. 2004). Van Goethem et al. (2019) recently reported that low doses of MLA improved the cognitive function of rats in an object recognition task, and MLA caused potentiation rather than inhibition of the activity of the endogenous agonist acetylcholine at the alpha7 nAChR. PHA-543613 is a potent and selective agonist of alpha7 nAChRs that has been characterized by rapid brain penetration and has been found effective in both auditory sensory gating and novel object recognition memory in rats (Wishka et al. 2006). Bali et al. (2019b) reported that PHA-543613 also exerts inhibitory effects on the firing activity of neurons similar to MLA which effect was explained by the rapid desensitization of nAChRs. According to the above pieces of evidence, in certain circumstances agonists and antagonists of the alpha7 nAChRs may have a similar net effect on neuronal function and/or cognition, which may provide a possible explanation for the currently observed interaction between memantine and PHA-543613 (Banerjee et al. 2005; Buccafusco et al. 2009).

In conclusion, our present results confirm that there is a beneficial pharmacological interaction between memantine and alpha7 nAChR ligands in terms of cognitive enhancement, both in the short-term and in the long-term memory domains. Also, our study provides further experimental evidence supporting the novel hypothesis on the role of the alpha7 nAChR in the mechanism of action of memantine. In the present report we also provided a detailed analysis of the pharmacological effects on different aspects of cognitive function, and showed that scopolamine, PHA-543613, and memantine differently affect spatial learning, short-term spatial memory and the recall of long-term memory in rats. The present findings may facilitate the understanding of cognitive enhancer effects of memantine, a known therapeutic agent as well as aid the development of more potent combinational therapeutic approaches against cognitive impairment.

## Supplementary Information

Below is the link to the electronic supplementary material.

Supplementary file1 (DOCX 81 kb)

## Data Availability

The datasets used and/or analyzed during the current study are available from the corresponding author on reasonable request.

## Abbreviations

AD: Alzheimer’s disease
ANOVA: Analysis of variance
LSD: Least significant difference
MLA: Methyllycaconitine
MWM: Morris water maze
nAChR: Nicotinic acetylcholine receptor
