# Supplementary material for: Potentiation of cognitive enhancer effects of Alzheimer’s disease medication memantine by alpha7 nicotinic acetylcholine receptor agonist PHA-543613 in the Morris water maze task
Source: Psychopharmacology (Berl). 2021 Aug 13;238(11):3273–81. doi: 10.1007/s00213-021-05942-4 (PMC8605977; doi:10.1007/s00213-021-05942-4)
Supplement: Supplementary file 1 — Supplementary file1 (DOCX 81 kb) [file 213_2021_5942_MOESM1_ESM.docx]

**
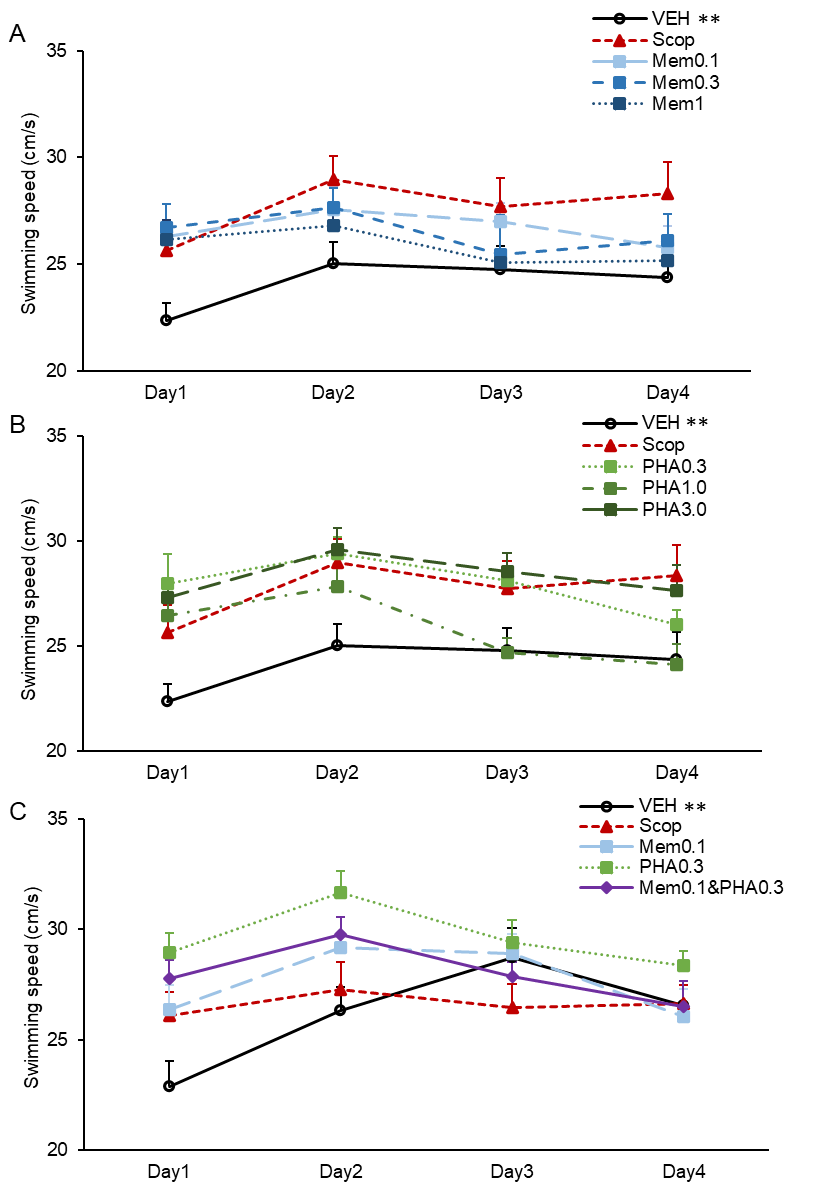
**

**Fig. S1** Effects of memantine (A) and PHA-543613 monotreatments (B) and their co-administration (C) on swimming speed in the water maze task. Swimming speed showed significant main effect of the treatments both in the dose-response experiments with memantine and PHA-543613 and in the co-administration study. Scopolamine accelerated the swimming of the animals, that was not affected by further treatments. Data are expressed as mean+SEM. Significant differences between a given treatment and scopolamine-only treatment were marked with asterisks: **p<0.01.
